# Perinatal Exposure to a Low Dose of Bisphenol A Impaired Systemic Cellular Immune Response and Predisposes Young Rats to Intestinal Parasitic Infection

**DOI:** 10.1371/journal.pone.0112752

**Published:** 2014-11-21

**Authors:** Sandrine Ménard, Laurence Guzylack-Piriou, Corinne Lencina, Mathilde Leveque, Manon Naturel, Soraya Sekkal, Cherryl Harkat, Eric Gaultier, Maïwenn Olier, Raphael Garcia-Villar, Vassilia Theodorou, Eric Houdeau

**Affiliations:** 1 Neuro-Gastroenterology and Nutrition INRA, UMR1331 Toxalim, Research Centre in Food Toxicology, Toulouse, France; 2 Intestinal Development, Xenobiotics & ImmunoToxicology INRA, UMR1331 Toxalim, Research Centre in Food Toxicology, Toulouse, France; 3 INRA, UMR1331 Toxalim, Research Centre in Food Toxicology, Toulouse, France; Baylor College of Medicine, United States of America

## Abstract

Perinatal exposure to the food contaminant bisphenol A (BPA) in rats induces long lasting adverse effects on intestinal immune homeostasis. This study was aimed at examining the immune response to dietary antigens and the clearance of parasites in young rats at the end of perinatal exposure to a low dose of BPA. Female rats were fed with BPA [5 µg/kg of body weight/day] or vehicle from gestational day 15 to pup weaning. Juvenile female offspring (day (D)25) were used to analyze immune cell populations, humoral and cellular responses after oral tolerance or immunization protocol to ovalbumin (OVA), and susceptibility to infection by the intestinal nematode *Nippostrongylus brasiliensis* (*N. brasiliensis*). Anti-OVA IgG titers following either oral tolerance or immunization were not affected after BPA perinatal exposure, while a sharp decrease in OVA-induced IFNγ secretion occurred in spleen and mesenteric lymph nodes (MLN) of OVA-immunized rats. These results are consistent with a decreased number of helper T cells, regulatory T cells and dendritic cells in spleen and MLN of BPA-exposed rats. The lack of cellular response to antigens questioned the ability of BPA-exposed rats to clear intestinal infections. A 1.5-fold increase in *N. brasiliensis* living larvae was observed in the intestine of BPA-exposed rats compared to controls due to an inappropriate Th1/Th2 cytokine production in infected jejunal tissues. These results show that perinatal BPA exposure impairs cellular response to food antigens, and increases susceptibility to intestinal parasitic infection in the juveniles. This emphasized the maturing immune system during perinatal period highly sensitive to low dose exposure to BPA, altering innate and adaptative immune response capacities in early life.

## Introduction

Endocrine-disrupting compounds (EDCs) encompass many agents of chemical or natural origin, able to imbalance hormone-driven processes in animals and humans. Among these products, bisphenol A (BPA) is ubiquitous in the environment, because of its intensive use in food packaging, including polycarbonate plastics and epoxy resins lining metal cans, as well as in thermal papers or flame retardants [1]. BPA has been shown to impact various physiological functions in animal models [2], including the immune system [3] that protects the organism from infections. According to the World Health Organization (WHO), infectious diseases are the third leading cause of death worldwide (http://who.int/mediacentre/factsheets/fs310/en/), and recent reports highlighted perinatal exposure to environmental EDCs as a cause of impaired host response capacity to infections [4],[5],[6],[7],[8],[9],[10],[11]. As BPA can be detected in human umbilical blood cord, amniotic fluid or maternal milk [12], a special attention has been paid to exposure during the perinatal period, during which most functions of the organism are immature, and considered as particularly vulnerable to adverse environmental factors, including EDCs. Accordingly, Luebke et al. compared the consequences of five xenobiotics on the immune system after perinatal or adult exposure, and concluded that perinatal exposure had more dramatic and long lasting adverse effects on immune system [13].

In the gut, the maturation process of the mucosal immune system is a continuous cascade that begins long before birth, and continues though early childhood. Indeed, the gut-associated lymphoid tissue (GALT) differentiates during fetal life, while at birth it undergoes further maturation with primary bacterial colonization and food, to achieve tolerance to luminal content including microbiota, and effective host defenses against pathogens. We previously showed that exposure of rats to BPA during gestation and lactation induced persistent deleterious effects on gut immune function in later life, exacerbating experimental inflammation in 2,4,6 TriNitroBenzene Sulfonic acid (TNBS)-induced colitis [14]. However, because these effects on the immune response in the colon have been investigated in adult individuals, long after the exposure of their dams to BPA, they did not depict the immune risk in young life, immediately following developmental exposure to BPA, which may be different or more severe from the effects observed later in adulthood. Whatever the species, the juvenile period is particularly challenging and critical for the developing immune functions. The weaning period in rodents represents a critical window characterized by modifications in both food and microbiota, with significant consequences on the immune system of the host for oral tolerance to luminal content, and defenses against foreign organisms (bacteria and parasites) [15]. During this time frame, it is suggested that innate and adaptive immune responses could be quantitatively and/or qualitatively different from normal responses, due to BPA-induced changes in the maturational process of both local (GALT) and systemic immune functions.

The present study was aimed at investigating the consequences of developmental exposure to a low dose of BPA on immune functions in juvenile rats aged of 25 days (D25), i.e. 4 days after weaning (D21) corresponding to the end of transmaternal BPA exposure. T cells and dendritic cells populations were analyzed, as well as the humoral and cellular response to an oral tolerance and immunization protocol with the food antigen ovalbumin (OVA). Furthermore, consequences of BPA exposure on susceptibility to infection were tested with the intestinal parasite *N. brasiliensis*. Due to gender-related differences in intestinal BPA effects in rats [14], our study was conducted in D25 females perinatally exposed through their dams at 5 µg/kg body weight (BW) per day of BPA, i.e. 10-fold below the US Tolerable Daily Intake for BPA (TDI, 50 µg/kg BW/d) currently set as the safety limit for human exposure. In January 2014, based on continuing uncertainties over the risks posed by the chemical, the European Food Safety Agency (EFSA) dropped the TDI to 5 µg/kg BW/d (http://www.efsa.europa.eu/en/press/news/140117.htm). We found that developmental exposure to BPA at this low dosage produced immunotoxicity in D25 offspring, and predisposed juvenile rats to intestinal parasitic infection.

## Material and Methods

### Animals and BPA treatment

Pregnant and lactating Wistar female rats (Janvier, France) were daily treated orally from 15th day of gravidity to weaning of pups (day 21) with 5 µg/kg BW/d of BPA, or the vehicle alone (4% ethanol in corn oil) as control group. Young offspring females rats aged of 25 days were assessed for oral tolerance and immunization to ovalbumin (OVA) and infection with the gut nematode *Nippostrongylus brasiliensis* (*N. brasiliensis*). They were kept at a constant temperature (22+/−1°C) and maintained on a 12∶12 h light/dark cycle (light on at 7h30 am) on corn pop bedding (Souralit, Spain). Food (Harlan Global Rodents Diets 2018, France) and tap water were available *ad libitum*. Estrogenicity of the feed has been previously measured as <20 pmol of estrogen equivalents per gram, considered as a negligible amount [16]. Cages and bottles were made of polypropylene to avoid any cross-contamination by BPA from environmental sources, and cages/water/bedding have been previously tested negligible for estrogenicity by using the E-Screen assay [16].

### Ethics approval

The animal experimental protocols were approved by the Comité d'Ethique de Pharmacologie-Toxicologie, Toulouse - Midi-Pyrénées (Registered as N°86 at the Ministry of Research and Higer Education, France); notification TOXCOM 0035/EH-2013, and conducted in accordance with the European directive 2010/63/UE.

### Experimental Protocol

#### Oral tolerance to ovalbumin

At 25 days of age, groups of female offsprings perinatally exposed to BPA, or vehicle (controls), were tested for oral tolerance induction (OVA-tolerized) and immunization (OVA-immunized) as indicated in Table 1. Briefly, at D25, all OVA-tolerized rats were gavaged with 20 mg OVA in 1 ml of 0.2 M bicarbonate buffer, pH 8.0, at day 0 (P0), while OVA-immunized rats were gavaged with bicarbonate buffer alone. One week later (P7, Table 1), all rats were primed subcutaneously with 100 µg OVA in Complete Freund Adjuvant (CFA), and boosted 2 weeks later (P21) by subcutaneous injection of 100 µg OVA. After 1 additional week (P28), rats were anesthetised and bled out at the abdominal aorta. Spleen and Mesenteric Lymph Nodes (MLN) were sampled for primary cell culture.

**Table 1:** Oral tolerance and immunization protocols.

Age of rats	D15 fetal life - D15	D25	D32	D46	D53
Day of protocol		P0	P7	P21	P28
**OVA-tolerized**	BPA 0 or 5 µg/kg of BW/day	Gavage OVA 20 mg in Bicarbonate buffer	OVA in CFA 100 µg sc	OVA boost 100 µg sc	Sacrifice Humoral and cellular response
**OVA-immunized**	BPA 0 or 5 µg/kg of BW/day	Gavage Bicarbonate buffer	OVA in CFA 100 µg sc	OVA boost 100 µg sc	Sacrifice Humoral and cellular response

CFA: Complete Freund Adjuvant; OVA: ovalbumin; sc: subcutaneous.

#### Infection with *Nippostrongylus brasiliensis*

Female rats aged of D25 peritanally exposed to BPA 5 µg/kg BW/d or vehicle (4% ethanol in corn oil) were used for nematode infection, as scheduled in Table 2. Briefly, D25 rats were infected subcutaneously (sc) with 1000 infective-stage larvae of *N. brasiliensis*. One week later (P7, Table 2), all rats were euthanatized, and blood and jejunal fragments were collected for bioplex, enzyme linked immunosorbent assay (ELISA), and Myeloperoxidase (MPO) enzymatic activity, a marker for neutrophil infiltration [17]. Feces were sampled for *N. brasiliensis* living larvae counting. Briefly, faecal content was collected and weighed, then water was added, and the mixture incubated in the dark at 28° for 1 hour. Charcoal powder was added, and the mixture was spread on moist filter paper, placed in *Petri* dishes containing water. The *Petri* dishes were incubated in the dark at 28°C for 7 days. Larvaes were collected by sedimentation. Living larvaes were counted under a microscope, and expressed in living larvae number/g feces.

**Table 2:** *Nippostrongylus brasiliensis* infection protocol.

Age of rats	D15 fetal life - D15	D25	D32	D39
Day of protocol		P0	P7	P14
**Procedures**	BPA 0 or 5 µg/kg of BW/day	sc injection with 1000 infective-stage larvae of *N. brasiliensis*	Euthanasia: Jejunum and blood sampling Feces cultures	Living larvae counting in feces cultures

### Measurement of Immunoglobulins (Humoral Response)

#### Anti OVA-IgG titers

Plasma anti-OVA IgG titers were measured using ELISA as follows: 96-well flat-bottomed plates (Nunc) were coated overnight with OVA (Sigma-Aldrich) in phosphate-buffered saline (PBS). After washes with PBS/0.05% Tween 20 (PBS-Tween), non specific binding sites were blocked with PBS/5% foetal calf serum (FCS). Plates were then incubated with plasma. Horse raddish peroxidase conjugated polyclonal rabbit anti-rat IgG (Abcam) was added. Finally, TMB substrate (Thermoscientific) added to each well. The reaction was stopped with 2N H_2_SO_4_ and plates read at 450 nm using an automatic Infinite M200 microplate reader (Tecan). Titers were expressed as ×10^3^ of the highest plasma dilution, giving an optical density at least twice the blank value.

#### Plasmatic IgE concentrations

IgE concentrations were measured in plasma of rats infected or not by *N. brasiliensis*, using Neo Biotech ELISA Kit (Cliniscience) according to the manufacturer's instructions.

### Measurement of spleen and mesenteric lymph nodes (MLN) Cells Activation

Spleens and MLN were removed and cells were isolated through a 40 µm nylon mesh in PBS/1% FCS.

#### Flow cytometry on day 25 splenocytes

Cells were stained with antibodies to the following markers: anti-CD4 (W3/25, Biolegend), anti-CD25 (OX-39, Biolegend), anti-foxp3 (FJK-16s, ebioscience), anti-CD11b (M1/70 BD Pharmingen), anti-CD103 (M290 BD Pharmingen), anti-MHCII (OX-6, Biolegend) and anti-CD172 (OX-41, Biolegend). Flow cytometry data collection was performed on MACSQuant Analyzers (Miltenyi Biotec). Data were analysed using VenturiOne (AplliedCytometry) software.

#### *In vitro* OVA cell restimulation

After washing, cells were seeded on 24-well plates at 2×10^6^ cells per well in Cerottini culture medium (Dulbecco modified Eagle medium without phenol red supplemented with 8% heat-inactivated FCS, 36 mg/l asparagine, 116 mg/l arginine, 10 mg/l folic acid, 1 g/l 4-[2-hydroxyethyl]-1-piperazineethanesulfonic acid, 0.05 mmol/l β-mercaptoethanol, 100 U/ml penicillin, 100 kg/ml streptomycin and 1 µg/ml fungizone) in the presence or absence of the specific antigen OVA 2 mg/ml. After 3 days of OVA stimulation, culture supernatants were collected and frozen at -80°C prior to cytokines measurement.

### Cytokines measurement

Cytokines were measured in supernatant of primary cell cultures of spleen and MLN, and on jejunal tissue segments. Frozen jejunal fragments were suspended in RIPA buffer (0.5% deoxycholate, 0.1% SDS and 1% Igepal in TBS) containing complete anti protease cocktail (Roche), and protein concentrations were measured using BCA optima kit (Interchim). IL13 and Growth-Regulated Oncogene/Keratinocyte Chemoattractant (GRO/KC) in jejunal tissues were measured using Bio-Plex Pro Assays kit (Biorad) following the manufacturer procedure, and results expressed in pg of cytokine per mg of protein loaded. Commercial ELISA kits (Duoset R&D Systems, Lille, France) were used for IFNγ, IL4, or IL10 cytokine measurements in primary cell culture supernatants or jejunal tissues. Data were expressed in pg of cytokine per ml of supernatant of cell culture media.

### Measurement of myeloperoxidase (MPO) activity in jejunal segments

Myeloperoxidase (MPO) activity was used as a marker for mucosal neutrophil infiltration [17]. Briefly, jejunal tissue segments were homogenized on ice in potassium phosphate buffer. After 3 cycles of freezing and thawing, suspensions were centrifuged at 10000xg for 15 min (4°C) and supernatants were discarded. Pellets were resuspended in the detergent hexadecyltrimethylammonium bromide buffer (Sigma) to release MPO from the primary granules. After sonication on ice and centrifugation (10000xg, 15 min, 4°C), supernatants were assayed spectrophotometrically for MPO activity. Results were expressed as MPO activity U/g of protein.

### Statistical Analyses

Statistical analyses were performed using the GraphPad Prism 4 software. All results are expressed as mean±SE. Comparison of means was performed using two ways Anova analysis with Bonferroni post-test or nonparametric Mann Whitney tests. A difference was considered significant when p<0.05.

## Results

### Effects of perinatal exposure to BPA in D25 rats on humoral response after oral tolerance protocol or immunization to OVA

**Figure 1:**
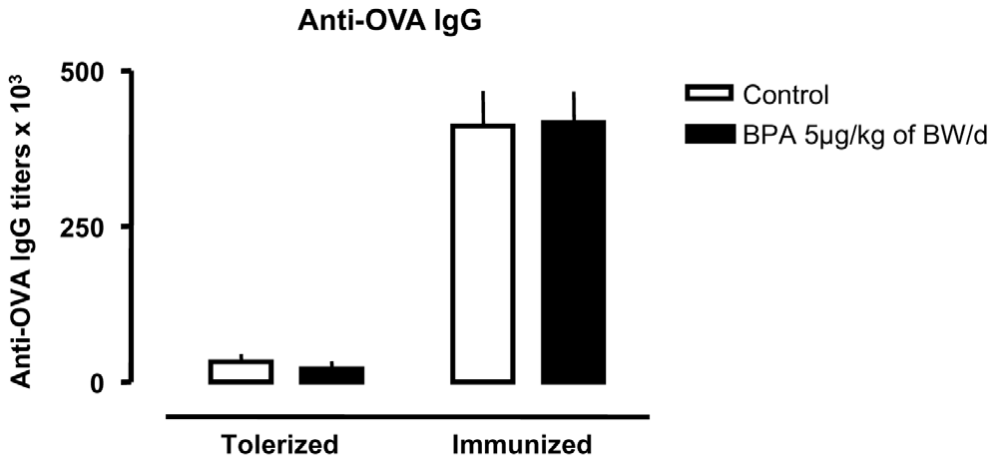
OVA-specific IgG titers in OVA-tolerized or OVA-immunized rats perinatally exposed or not to 5 µg/kg of BW/d of BPA. Tolerization was achieved in all groups, as attested by a significant decrease of anti-OVA IgG titer in OVA-tolerized rats compared to OVA-immunized rats. N = 14 to 17 rats per group.

Oral tolerance is manifested by the absence of humoral response (anti-OVA IgG) to the orally administered antigen (OVA). In control D25 female rats, oral OVA prior to OVA systemic immunization significantly decreased by 50-fold anti-OVA IgG titers, validating our protocol of oral tolerance (Figure 1). Perinatal exposure to BPA did not change anti-OVA IgG titers in OVA-tolerized rats nor -immunized rats when compared to non-exposed controls. Using this protocol, no anti-OVA IgE response occurred excluding any allergic response (data not shown).

### Perinatal exposure to BPA impaired cellular response in spleen and MLN of OVA-tolerized and -immunized D25 rats

**Figure 2:**
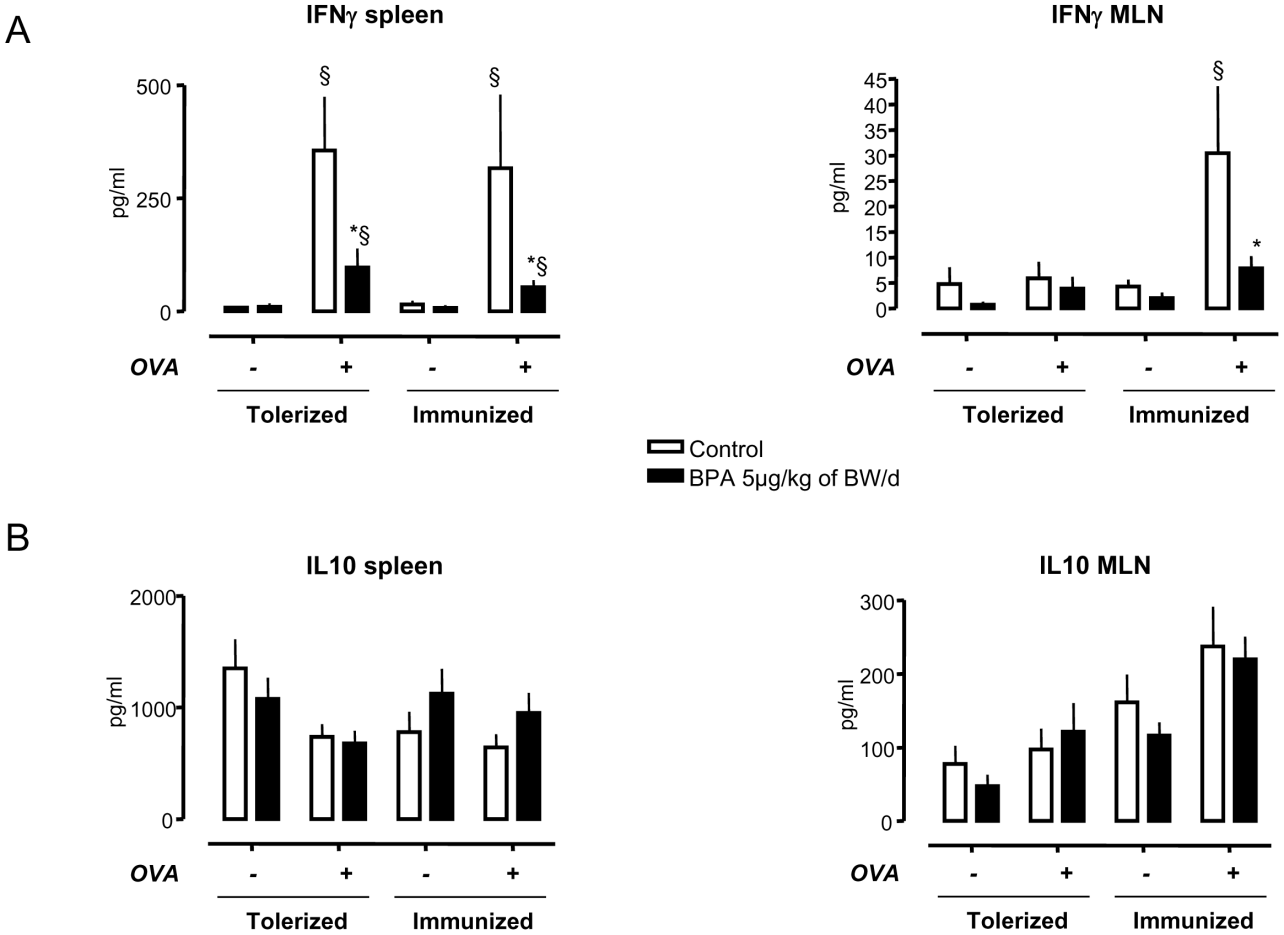
OVA-specific cytokine profile (IFNγ and IL10) in cell culture supernatant of spleen and MLN cells of rats perinatally exposed to BPA (5 µg/kg of BW/d) or not (controls). (A) After OVA restimulation, cultured splenocytes from rats perinatally exposed to BPA showed a significant decrease of IFNγ secretion in OVA-tolerized and -immunized conditions compared to non-exposed controls to BPA. In MLN cell culture, perinatal exposure to BPA induced a significant decrease of IFNγ in response to OVA restimulation in OVA-immunized conditions only when compared to controls. (B) No difference in IL10 concentrations was observed in this experiment. * p<0.05 compared to control, and § p<0.05 compared to basal conditions without OVA stimulation. N = 12 to 14 rats per group.

Oral tolerance is not only manifested by the absence of humoral response (anti-OVA IgG), but also of immune cellular response to the orally administered antigen (OVA). Spleen and MLN cells were isolated from OVA-immunized and OVA-tolerized rats, with and without perinatal exposure to BPA. Cellular response to OVA was assessed by analyzing cytokine concentrations (IFNγ and IL10) in the supernatant of splenocytes and MLN cells after *in vitro* OVA restimulation (Figure 2). Perinatal exposure to BPA did not affect basal IFNγ concentration (i.e. without OVA restimulation) in splenocytes or MLN supernatants from either OVA-tolerized or OVA-immunized rats compared to their respective controls non-exposed to BPA (Figure 2A). In control rats with OVA-tolerization or -immunization, *in vitro* OVA stimulation induced IFNγ production by splenocytes (p<0.05). In contrast, perinatal exposure to BPA led to a sharp decrease in the *in vitro* OVA-induced IFNγ release by splenocytes compared to non-exposed controls with OVA-tolerization or -immunization (Figure 2A).

In MLN cells, *in vitro* OVA restimulation induced a significant increase of IFNγ concentration production in control OVA-immunized rats only, while BPA exposure significantly decreases by 3-fold the OVA-induced IFNγ secretion in these animals (p<0.05). No significant change in IL10 secretion was observed in rats perinatally exposed to BPA compared to their controls (Figure 2B).

### Perinatal exposure to BPA decreased T lymphocyte and dendritic cell populations in the spleen and MLN

**Figure 3:**
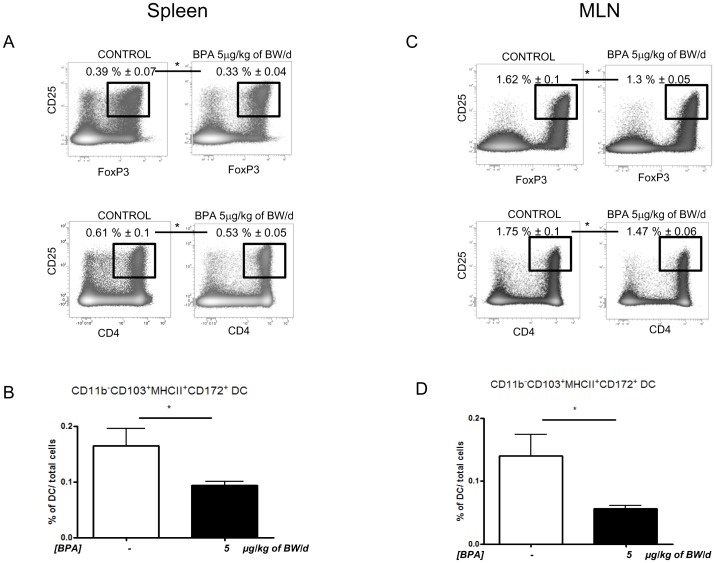
Effect of perinatal exposure to BPA (5 µg/kg of BW/d) on T lymphocytes and dendritic cells populations in spleen and MLN. (A) D25 rats perinatally exposed to BPA showed a decrease of T regulatory CD4^+^CD25^+^Foxp3^+^ and of T helper CD4^+^CD25^+^ lymphocytes (A, C), as well as of CD11b^−^CD103^+^MHCII^+^CD172^+^ dendritic cells (B, D) in spleen and MLN. * p<0.05 compared to controls. N = 12 rats per group.

Perinatal exposure to BPA significantly decreased (p<0.05) the percentage of Treg (CD4^+^ CD25^+^ Foxp3^+^) and T helper (CD4^+^ CD25^+^) lymphocytes in the spleen and MLN of D25 rats relative to controls (Figure 3A and C). Furthermore, BPA treatment not only induced a decrease in T cell populations, but also a drop in CD11b^−^CD103^+^MHCII^+^CD172^+^ dendritic cells (p<0.05) (Figure 3B and D).

### Rats perinatally exposed to BPA developed susceptibility to *Nippostrongylus brasiliensis* intestinal infection

**Figure 4:**
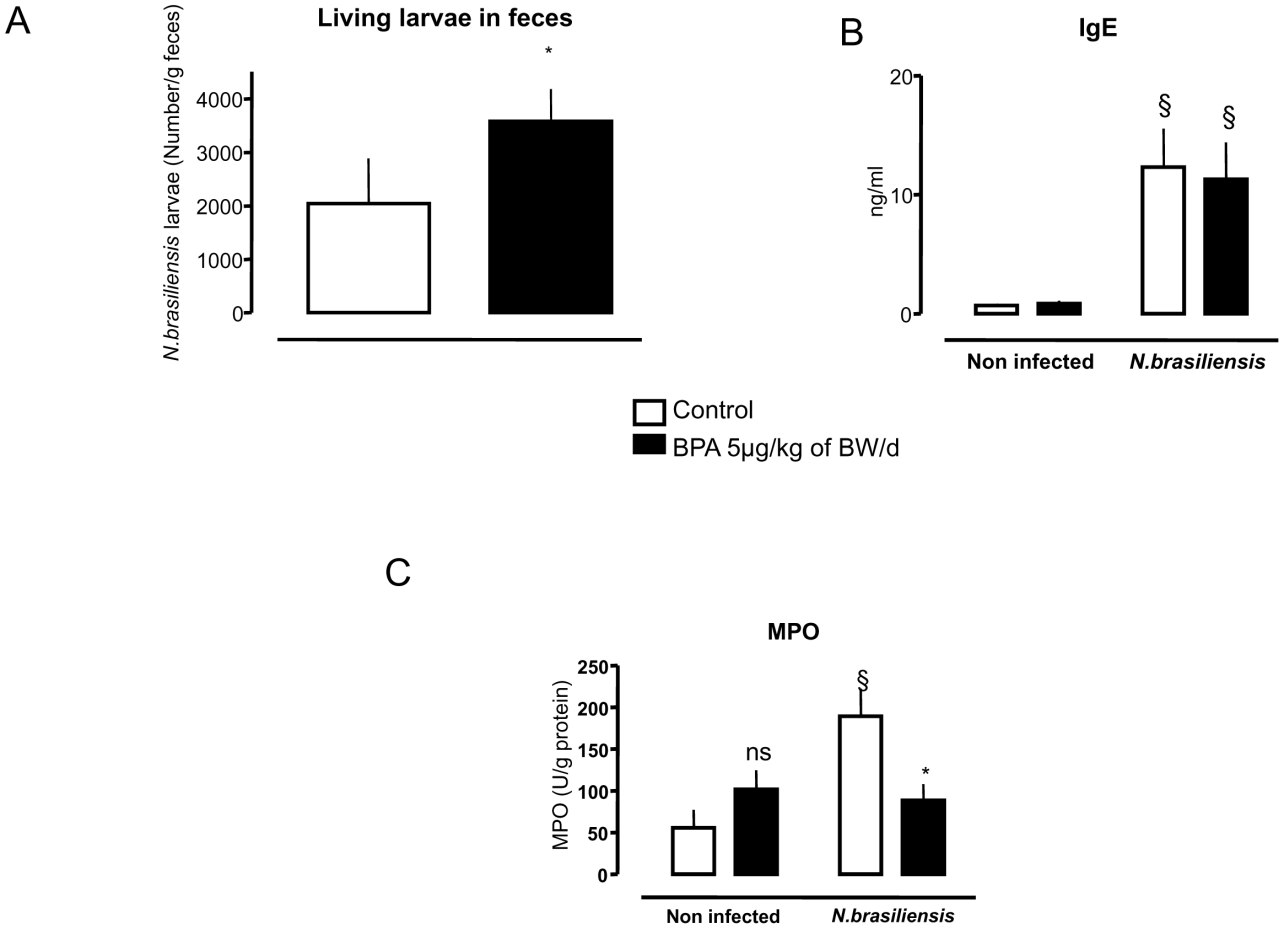
Effects of perinatal exposure to BPA (5 µg/kg of BW/d) on gut parasitic infection by *Nippostrongylus brasiliensis* (*N. brasiliensis*). D25 rats perinatally exposed to BPA showed an increased number of living larvae in feces 7 days after infection compared to controls (A), without changes in plasmatic IgE concentrations (B). Furthermore, BPA-exposed rats displayed a significant decrease in MPO activity in the jejunum following *N. brasiliensis* infection (C). * p<0.05 relative to controls, and § p<0.05 compared to respective non-infected rats. N = 7 to 8 rats per group.

The Figure 4 shows that rats peritanally exposed to BPA, and infected at D25 with *N. brasiliensis*, displayed a 1.5 fold increase of living larvae number in their feces compared to controls (Figure 4A). This increase was not associated with change of plasmatic total IgE concentrations (Figure 4B), but found related to a sharp decrease of jejunal MPO activity (p<0.05), indicating a decreased neutrophil infiltration compared to control rats with nematode infection (Figure 4C).

**Figure 5:**
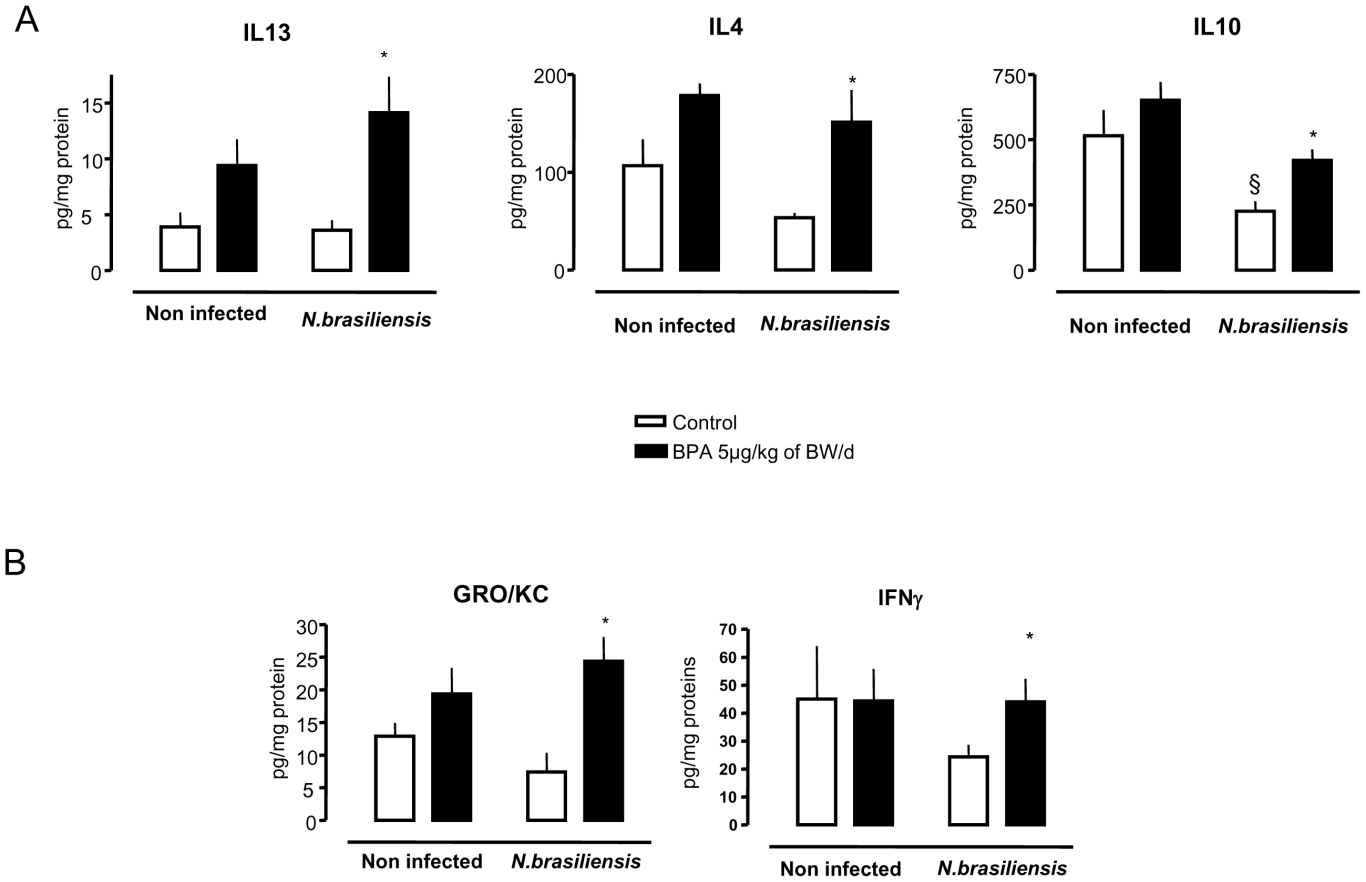
Effects of perinatal exposure to BPA (5 µg/kg of BW/d) on cytokine profile in the jejunum of infected rats with *N. brasiliensis*. (A) Th2 cytokine levels: IL13, IL4 and IL10, and (B) pro-inflammatory cytokines: GRO/KC and IFNγ. * p<0.05 relative to controls, and § p<0.05 compared to respective non-infected rats. N = 7 to 8 rats per group.

Despite no change in IgE response (Figure 4B), *N. brasiliensis* infection in rats peritanally exposed to BPA significantly increased Th2 cytokines (IL13 and IL4), anti-inflammatory (IL10), as well as the pro-inflammatory cytokines GRO and IFNγ in the small intestine (jejunum) compared to infected control rats (Figure 5A and B).

## Discussion

Bisphenol A is a common environmental EDC whose deleterious effects on immune functions have been reported in adult animals after perinatal exposure [14],[18]. In this study, we elucidated the early consequences on both immune system and host defense in young rats at the end of the period of exposure to a low dose of BPA through their dams. We report that juvenile rats perinatally exposed to BPA are able to set up a normal humoral response toward food antigens in early post-weaning days, but failed to induce a proper cellular immune response after systemic immunization, indicating an immunosuppressive effect. Furthermore, perinatal exposure to BPA increased susceptibility to *N. brasiliensis* parasitic infection by deregulating Th1/Th2 cytokines profile in infected intestinal mucosa.

A consensus exists that the effects of BPA on the host immune status depend on various parameters, including the period of exposure (adult *versus* perinatal), sex and animal model (Th1 or Th2 skewed animals), as well as the dose and route used for BPA treatment [3]. For instance, in a rat model of TNBS-induced colitis, perinatal exposure to BPA from mid-pregnancy to lactation exacerbated mucosal inflammation at adulthood, suggesting immunoactivation, whereas a direct exposure in adults to the same dosage dampened the inflammatory response in the colon [14]. This underlines the age-sensitivity of the immune system to EDC exposure, and the fact that the perinatal life is a critical window for BPA perturbations on the developing gut immune system, as observed with other environmental stimuli [19],[20]. In the current study, we reported that perinatal exposure to 5 µg/kg of BW/d of BPA had no effect on anti-OVA IgG titers in juvenile offspring rats after oral tolerance or immunization protocols. This is in accordance with a previous study with OVA TCR mice (a transgenic model favouring OVA recognition) perinatally exposed to BPA, and using a similar tolerance protocol with high OVA dosage [21]. Interestingly, our findings in juvenile rats are in contrast with the situation reported at adulthood (i.e. at D45), when rats perinatally exposed to the same BPA dosage displayed an increased anti-OVA IgG titers OVA after tolerization and immunization protocols [22]. A discrepancy in BPA effects among ages has been reported in recent human epidemiological studies, when high BPA urinary levels were found associated with higher serum cytomegalovirus antibody levels (considered a marker of altered cell-mediated immune function) in a cohort of above 18-year old people, whereas a negative correlation was noticed for people under 18-years of age [23].

In the present study, although no change in OVA-specific antibody production was observed in D25 rats perinatally exposed to BPA, a BPA-induced suppressive effect on IFNγ production by splenocytes and MLN cells occurred after OVA stimulation *in vitro* in OVA-immunized rats. A similar effect of BPA was also observed in OVA-tolerized rats, except in MLN where OVA stimulation did not induce any IFNγ production over basal levels. An inhibition of cytokine secretion in response to antigen stimulation is in agreement with a marked decrease of T (regulatory and helper) and dendritic cells in basal conditions in the spleen and in the MLN of rats perinatally exposed to BPA. To our knowledge, the present study is the first to show that dendritic cells are a target for perinatal BPA effects, and that exposure to BPA at a low dose *in utero* and during lactation induced a general decrease of cellular response in early life, indicating systemic immunosuppression.

It is well established that the maturing period of the immune system in young ages, when faced with multiple environmental challenges, is a vulnerable period to luminal aggressors, and at high risk for the development of infectious diseases. We hypothesized that the BPA-induced immunosuppressive effect could have adverse outcome when inflammatory response is critical, for example, in pathogen clearance. For instance, perinatal exposure to BPA increased susceptibility to *Leishmania major* infection in mice, attesting by higher footpad swelling, and production of IL4 and IFNγ by splenocytes [18]. In the present study, we addressed the consequences of perinatal exposure to BPA on intestinal infection with the nematode *N. brasiliensis* in juvenile rats. After subcutaneous infection, nematode larvae migrate into the lungs, and hence reach the intestine via the trachea where they mature to the adult stage [24]. The early stages of infection include neutrophil recruitment in the intestinal mucosa, and their accumulation at infection sites participating in failure of infective larvae to establish and mature into adults in the gut. Indeed, the enzyme MPO, located in neutrophil primary granules, produces hypohalous acids known as central to the microbicidal activity of neutrophils into the mucosa [25]. We observed that perinatal BPA exposure sharply decreased MPO activity in the jejunum after *N. brasiliensis* infection, reflecting an inhibition of neutrophil accumulation at infected sites. *N. brasiliensis* infection is well known to suppress Th1 immune response, and to promote Th2 polarization [26],[27]. Neutrophils contribute to the development of an optimal Th2 response, by eliminating parasite-associated bacteria that otherwise induced a Th1-type response [28]. The deleterious effect of BPA on neutrophil killing activity might be responsible for the increase of the Th1 cytokine IFNγ, and a delayed clearance of the parasite. This is in accordance with results from Pesce et al. demonstrating that neutrophils depletion in *N. brasiliensis*-infected mice led to increased IFNγ production, and decreased Th2 responses, hence favoring a delayed worm expulsion [28]. In our study, we hypothesized that BPA-induced depletion in neutrophils, and of associated MPO microbicidal activity, may be responsible for the increase of IFNγ only observed in infected rats, thus contributing to failure in the gut clearance of the nematode.

We also examined the Th2 cytokines profile in the jejunum of both BPA-exposed rats and non-exposed controls infected with *N. brasiliensis*. In control rats, the low levels (IL4 and IL13) or even the decrease (IL10) of Th2 cytokines in the infected mucosa is consistent with the fact that young rodents (≤4 weeks) displayed a weakened systemic type 2 immune response when compared to *N. brasiliensis*-induced cytokine production in adults, as reported in mice [29]. Although similar plasmatic IgE levels were observed between controls and BPA-exposed rats infected by *N. brasiliensis*, a significant increase of IL4 in jejunal mucosa was observed only in BPA-exposed rats. After helminthic infection, IgE production is regulated by both IL4 and IFNγ [30]. Indeed, administration of anti-IL4 or anti-IL4 receptor, or of the recombinant IFNγ inhibits IgE production in mice infected by intestinal nematode parasites, such as *N. brasiliensis* or *Heligmosomoides polygyrus* [31],[32],[33],[34]. Furthermore, Uchikawa et al. demonstrated that IgE production in *N. brasiliensis* infected rats was associated with a concomitant suppression of IFNγ production/secretion [26]. In our study, because IFNγ increased concomitantly with IL4 production, it is suggested that the antagonistic effect of IFNγ could counterbalance the IL4-induced IgE production, and explain why plasmatic IgE levels in BPA-exposed rats remained unchanged despite higher IL4 production. In addition, recombinant IFNγ increased the fecundity of adult *N. brasiliensis* [27], and impaired the protective immunity towards the parasite [26],[27]. Consequently, the observed increase of living larvae in the feces of rats perinatally exposed to BPA might be a consequence of an enhanced IFNγ production at local site. In addition, the peak of *N. brasiliensis* eggs is always observed 7 days after inoculation, and sharply decline in the following days [27]. As we observed a dramatic increase of living larvae in the feces of BPA-exposed rats one week after *N. brasiliensis* infection, we concluded that perinatal exposure to a low dose of BPA impaired the host protective immunity toward nematodes in early offspring life, resulting in an increase of parasite burden and delayed intestinal clearance of adult worms.

In conclusion, the present study provides evidence that the developing immune system throughout the perinatal period is vulnerable to a low-dose exposure to BPA. Adverse outcomes in juvenile rats include an impaired sensitization to dietary antigen proteins (i.e. systemic immunosuppression), and increased susceptibility to enteric parasitic infection. These findings are of particular interest as oral tolerance, immunization/vaccination and infections are more frequent in young individuals compared to adults. Further studies on the developmental impact of BPA on the immune system may help to investigate EDCs as potential primary contributors to infectious diseases.
